# Ancillary Data Record Linkage to characterize the completeness of data for the All of Us Research Program.

**DOI:** 10.23889/ijpds.v7i3.2090

**Published:** 2022-08-25

**Authors:** Yuyang Yang, Kelsey Rodriguez, Melissa Basford, Sidd Nambiar, Lew Berman, Sidd Nambiar

**Affiliations:** 1 Northwestern University; 2 Vanderbilt University; 3 National Institute of Health

## Objectives

The All of Us Research Program (AoURP) is an ambitious effort to gather health data from one million Americans to accelerate research. We linked Electronic Health Records (EHR) and insurance claims data to characterize the degree to which ancillary datasets can improve data completeness for care received by AoURP participants.

## Approach

We sought to link EHR data for 400,000 consented AoURP participants with insurance claims data provided by IPM.AI (Swoop Analytics), a commercial analytics company who have insurance claims data for 300M (over 90% of) Americans. We utilized a HIPAA-compliant privacy-preserving record linkage method (tokenization, provided by Datavant) to match patients between datasets. We evaluated match fidelity and the degree of overlap between AoURP EHRs and IPM.AI claims data. We characterized the association of patient and organizational level factors (demographics, healthcare provider organization, reporting site) with match performance.

## Results

As of submission of this abstract, 41% of AoURP EHRs matched with IPM.AI claims. We compared patient healthcare encounters, diagnosis codes (DX), procedure codes (PX), and national drug codes (NDC) for matched patients by month. The union of AoU and IPM.AI data greatly increased data completeness in matched patients. Only 20% of healthcare encounters were seen by AoURP and IPM.AI concurrently while 25% were unique to AoU EHRs and 55% to IPM.AI claims on a monthly level. The number of diagnosis events compared between AoURP and IPM.AI is roughly equal (AoU +6%) while procedure events are elevated in claims data (23%) and drug counts are greatly elevated in AoURP EHR data (71%). We found that matched patients had more healthcare encounters compared to unmatched patients.

## Conclusion

To our knowledge this is the first effort to address challenges in AoURP data completeness through complementary data linkage. Our results suggest that supplementary data linkage can improve data completeness in a large national research initiative. We identified several patient factors that require further investigation in improving match fidelity.

